# Synthesis of New Nitrofluoroquinolone Derivatives with Novel Anti-Microbial Properties against Metronidazole Resistant *H. pylori*

**DOI:** 10.3390/molecules22010071

**Published:** 2017-01-04

**Authors:** Luay Abu-Qatouseh, Mohammad Abu-Sini, Amal Mayyas, Yusuf Al-Hiari, Rula Darwish, Talal Aburjai

**Affiliations:** 1 Faculty of Pharmacy, University of Petra, 11914 Amman, Jordan; 2 Faculty of Pharmacy, University of Jordan,11914 Amman, Jordan; mohammad.abusini@zuj.edu.jo (M.A.-S.); hiary@ju.edu.jo (Y.A.-H.); rulad@ju.edu.jo (R.D.); aburjai@ju.edu.jo (T.A.); 3 Faculty of Pharmacy, Al-Zaytoonah University of Jordan, 11733 Amman, Jordan; 4 Faculty of Health Sciences, American University of Madaba, 11821 Madaba, Jordan; a.mayyas@aum.edu.jo

**Keywords:** nitrofluoroquinolones, *H. pylori*, urease inhibition

## Abstract

One of the major therapeutic approaches to preventing relapse and accelerating the healing of duodenal and gastric ulcers is the eradication of *Helicobacter pylori*. Due to the emergence of antibiotic resistance among clinical strains of *H. pylori*, alternative approaches using newly discovered antimicrobial agents in combination with the standard regimens for the treatment of *H. pylori* are increasingly needed. The purpose of the present study was to investigate the effect of newly synthesized 8-nitroflouroqunolone derivatives when used either alone or when combined with metronidazole against metronidazole-resistant *H. pylori*. Based on the standard antimicrobial susceptibility testing methods and checkerboard titration assay, all of the tested compounds showed interesting antimicrobial activity against 12 clinical strains of *H. pylori*, with the best in vitro effect for compound **3c**. In addition, synergistic and additive activities of some of the tested compounds were observed when combined with metronidazole. Furthermore, among the tested nitroflouroquinolone derivatives, compound **3b** showed significant urease inhibition activity with IC_50_ of 62.5 µg/mL. These results suggest that 8-nitroflouroquinolone derivatives may have a useful role in combination with anti-*H. pylori* drugs in the management of *H. pylori*-associated diseases.

## 1. Introduction

*Helicobacter pylori* has been increasingly emerging as a major cause of chronic gastritis and peptic ulcer worldwide [1,2]. Furthermore, *H. pylori* colonization of the gastric mucosa constitutes a major risk factor in the pathogenesis of gastric cancer and gastric mucosa-associated lymphoid tissue (MALToma) [3,4,5]. The eradication of *H. pylori* and/or attenuating its associated virulence factors can therefore contribute to improving the clinical conditions of patients infected with this bacterium, including the acceleration of peptic ulcer healing and minimizing the recurrence of gastric cancer [6]. Current approaches for the effective eradication of *H. pylori* include the use of at least two antibiotics and a proton pump inhibitor (triple and more recently quadruple therapy) [7,8]. However, due to the emergence of antibiotic resistance among *H. pylori* clinical strains—particularly against metronidazole and clarithromycin—higher rates of treatment failure of triple therapy have been reported [9,10]. Therefore, a search for new approaches with high efficacy and safety against *H. pylori* infection is necessary.

Since their discovery, fluoroquinolones have been extensively considered as major successful therapeutic antibacterial and anticancer agents [11,12,13,14]. However, a low level of resistance against fluoroquinolones among *H. pylori* clinical strains has been reported [15,16]. In a previous work, the biological activities of a group of 8-nitrofluoroquinolone derivatives have been evaluated, and strong antibacterial effect against *Escherichia coli* and *Staphylococcus aureus* was observed [17,18].

In general, the synthesis of novel C-7-substituted derivatives of 1-cyclopropyl-6-fluoro-8-nitro-4-oxo-1,4-dihydroquinoline-3-carboxylic acid having an 8-nitro substituent as an electron withdrawing group was evaluated for biological activity. In the present study, the antibacterial activity of previously described 8-nitrofluoroquinoline derivatives and two newlysynthesized 8-nitrofluoroquinolone derivatives was tested against twelve clinical strains and one control strain of *H. pylori* which are resistant to metronidazole. In addition, the effect of the potentially effective derivatives in combination with metronidazole against metronidazole-resistant *H. pylori* strains was evaluated.

## 2. Results and Discussion

### 2.1. Synthesis of New Compounds

**Scheme 1:**
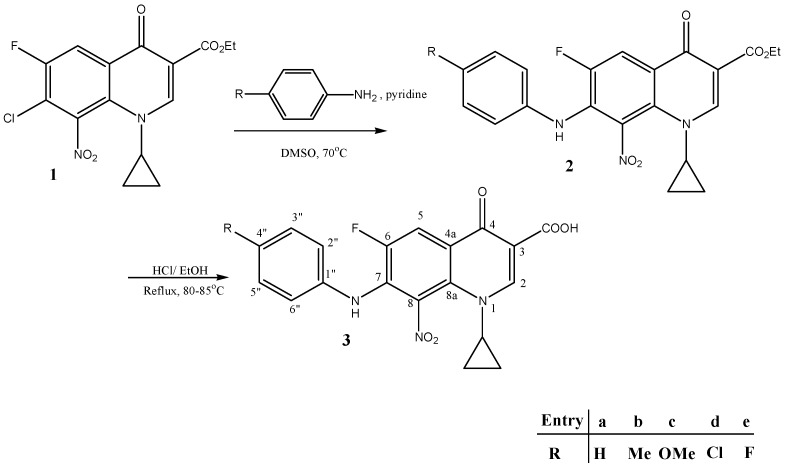
General procedure for the synthesis of novel target compounds **3**(**a**–**e**). DMSO: dimethylsulfoxide.

Synthesis of ester **1** was conducted following a previously reported procedure with good yields [18]. Compounds **2a**–**e** were synthesized by the reaction of substituted anilines with compound **1** under reflux (Scheme 1). Methoxy **2c** was obtained in higher yields compared to other derivatives.Hydrolysis of nitro ester **2** generated nitro acid target **3**. Compounds **2** and **3a**–**e** were identified and characterized by infrared spectroscopy (IR), mass spectrometry (MS), and ^1^H- and ^13^C-NMR spectroscopic analyses. The data for **2b**,**c** and **3b**,**c** are presented in the experimental section, since they were the most active.

The ^1^H-NMR spectra of all synthesized compounds (**3a**–**e**) contained a doublet for H-5 (3*J*_H-F_ = 10–13 Hz) at ~8.0 ppm. The splitting of this signal was caused by the vicinal fluorine, and indicated the presence of the fluoroquinolone nucleus in all of these compounds. Similarly, the singlet for H-2 at ~9.0 ppm effectively confirmed that compound **3** had been successfully formed. The ^1^H-NMR spectra of the side chain of **3a**–**e** contained new singlets in the range of 2.3–3.7 ppm and broad singlets in the range of 6.5–8.7 ppm, which were assigned to the aromatic methyl or methoxy and NH, respectively. The appearance of new peaks in the ranges of 6.8–7.2 ppm corresponding to the aromatic side chain protons was further proof. These signals effectively confirmed that the aryl amine side chains had been effectively incorporated into compound **3**. The appearance of a new peak above 12 ppm in **3** accompanied by the parallel disappearance of the triplet, quartet pattern for ester derivative **2** indicated the success of the hydrolysis step.

All of the carbons belonging to arylamine side chains were recognizable by their number, position, and orientation in depth charts in the aliphatic region. These signals confirmed that the arylamine chains had been successfully incorporated in compounds **3a**–**e**, further confirmation was noticed by the appearance of methyl and methoxy peaks within aliphatic regions. For the fluoroquinolone part, the ^13^C-NMR spectra of compounds **3a**–**e** contained a doublet (1*J*_C-F_ = 250 Hz) at ~150 ppm for C-6, which indicated the presence of the fluoroquinolone (FQ) nucleus in all of these compounds. This was also confirmed from depth analysis. The splitting of the neighboring carbon signals at C-5 and C-7 into doublet peaks in these compounds (2*J*_C-F_~20 Hz) effectively confirmed that they were all vicinal to a fluorine atom.

High- and low-resolution mass analysis was carried out for most compounds, and confirmed the expected compounds. (M + 1) molecular peak was obtained in most cases. Some cases had witnessed the appearance of (M + 2) molecular peaks due to bad tuning of the detector. This phenomenon can be also referred to the presence of multiple nitrogen atoms of the expected compounds. Positive mode of ionization adopted in the analysis procedure renders nitrogen atoms in their protonated form. Infrared spectroscopy was carried out for all compounds. Characteristic peaks of carbonyl, hydroxyl, amino, N=N, and C-X confirmed the identity of the expected compounds.

### 2.2. Antimicrobial Activity against Metronidazole-Resistant *H. pylori*

Previous work on the antimicrobial activity of 8-nitrofluoroquinolones reported variation in the potency of the tested compounds against Gram positive and Gram negative bacteria. It has been suggested in our group that the variable activity of these compounds could be related to the hydrophobic/hydrophilic properties of their structures, with no specific pattern [17,18].

*H. pylori* possesses unique biological characteristics among all eubacteria. Being a microaerophilic slow growing microorganism would dictate different activities of the antimicrobial agents against this pathogen, particularly in vivo. In this study, 8-nitrofluoroquinolone derivatives were evaluated for their antimicrobial properties against clinical strains of *H. pylori*. The initial screening showed that all of the tested compounds had antimicrobial activity against *H. pylori*, with a potent effect of compound **3c** (Table 1). The minimum inhibitory concentration (MIC) values of the tested compounds against the clinical strains were in the range of 2–64 µg/mL, which are higher than ciprofloxacin (Table 2). The best activity was reported for compound **3c**, followed by compounds **3a** and **3d**, respectively.

In terms of the antimicrobial effects of the combinations of the tested compounds with metronidazole against the metronidazole-resistant strains, our study reported interesting synergistic activity of the metronidazole **3a** and metronidazole **3d** combinations with fractional inhibitory concentration (FIC) indices of 0.328, although these compounds when tested alone showed moderate activity against the tested strains. It could be suggested that these compounds might work on the cell membrane of *H. pylori*, making it more permeable to metronidazole, since these compounds have some hydrophilic properties. Metronidazole is reduced to disrupt energy metabolism of anaerobes by hindering the replication, transcription, and repair process of DNA results in cell death (i.e., it inhibits nucleic acid synthesis by disrupting the DNA of microbial cells). Although metronidazole is a low molecular weight compound that diffuses across cell membranes, facilitating its entry and achieving an adequate concentration at its site of action would result in better effects on the bacteria, and this is done by using compoundswhich have some hydrophilic properties. Moreover, the combination of metronidazole + **3c** and metronidazole + **3e** showed an additive effect, with FIC indices of 0.656 and 0.874, respectively (Table 3). The combination of metronidazole + **3b** showed indifferent activity with FIC indices of 1.312, providing that compound **3b** reported the least antimicrobial effect against the studied strains when tested alone.

### 2.3. Inhibitory Effects of the Synthesized Compounds against H. pylori Urease Enzyme

One of the new potentials for the control of *H. pylori* infections is to reduce its pathogenicity by targeting its virulence factors [19]. The urease enzyme is a major virulence factor of *H. pylori* required for the optimal colonization and survival of this pathogen in the acidic conditions of the human stomach [20]. In this study, 8-nitrofluoroquinolones were evaluated for their potential inhibitory effect against *H. pylori* urease. The compounds were tested for their inhibitory effects in the range of 0.5–500 µg/mL by the standard colorimetric method mentioned above, and the compounds that showed less than 60% inhibition were considered to have no significant effect [20]. Our work showed that significant urease inhibition effect was reported only for compounds **3b** and **3c**, with higher inhibitory effect for **3c** with IC_50_ value of 62.5 µg/mL (Table 4). This unique inhibitory effect of the methyl and methoxy derivatives might be due to allosteric action against the enzyme, and needs to be further investigated.

### 2.4. Structural Activity Relationship Studies

The results of this research reveal that **3c** (para methoxy aniline substituents) exhibited significant inhibitory activity against *H. pylori*. An attempt was carried out to understand the underlying structural effect of these compounds. Esters **1** and **2c** were both evaluated for their antimicrobial properties against clinical strains of *H. pylori*, demonstrating no activity. At this early stage of research, we can suggest that activity of these FQs (**3a**–**c**) lies mainly within the 4-oxo-pyridine-3-carboxylic acid system. Losing the free COOH group in **2** condensed the activity significantly. It was noticed that ester **2c** is higher in its activity than **1**, which lacks any substituent at C7—although both have low activity against *H. pylori*. This finding designates that increasing lipophilicity through aniline substituents at C-7 can significantly improve the activity against *H. pylori*. It is well known and was previously highlighted by our group that highly lipophilic fluoroquinolone ligands improve antibacterial activity against Gram positive bacteria in particular, and to some extent against Gram negative bacteria Therefore, the activity of FQs **3a**–**e** could be explained to some extent by their high lipophilicity [17,18]. On the other hand, our group has emphasized that Gram negative activity was boosted through C-7 and C-8 hydrogen bond network of fluoroquinolone systems. The activity of compounds **3a**–**e** against Gram negative *H. pylori* could also be attributed also to the extra hydrogen bond network that might be formed on C-7 (NH) and C-8 (NO_2_) groups in our FQs. The extraordinary activity of compound **3c** in particular against *H. pylori* can be explained by the additional H-B displayed by the para-methoxy group oxygen in the anisidine substituent of **3c**. Again, same structural features of **3c** and extra H-B of the methoxy can illuminate its unique inhibitory activity against *H. pylori* urease enzyme.

Fluoroquinolones **3a**–**e** are derivatives of clinically used antibacterial quinolones such as the drug ciprofloxacin. They exhibit the same nucleus—1-cyclopropyl-6-fluoro-4-oxo-1,4-dihydroquinoline-3-carboxylic acid with aniline substituent instead of piperazine at C-7. Infact, the aniline system is known to be used in many drug interties as an analgesic drug. Therefore, we do expect them to suffer similar side effects and toxicity to known commercial FQs.

## 3. Materials and Methods

### 3.1. Materials and Instruments

All chemicals, reagents, and solvents were of analytical grade and used directly without further purification. *p*-anisidine and *p*-toluidine were purchased from Fluka (Buchs, Switzerland). Reducing agent—anhydrous stannous chloride crystals—was purchased from Fluka (Buchs, Switzerland). Sodium nitrate was purchased from Sigma Aldrich (St. Louis, MO, USA). Melting points (mp) were determined in open capillaries on a Stuart scientific electro-thermal melting point apparatus (Stuart, Staffordshire, UK) and are uncorrected. Thin layer chromatography (TLC) was performed on 10 × 10 cm^2^ aluminum plates pre-coated with fluorescent silica gel GF254 (ALBET, Germany), and was visualized using UV lamp (at 254 nm wavelength/short wavelength/ long wavelength). Mobile phase mixtures were: 94:5:1 chloroform–methanol–formic acid (CHCl_3_–MeOH–FA) (system 1) and 50:50 (*n*-hexane–ethyl acetate) (system 2). Nuclear magnetic resonance spectra were recorded on a Varian Oxford-300 (300 MHz) spectrometer (ALT, CO, USA), and a Bruker, Avance DPX-300 spectrometer, a Bruker Avance-400 (400 MHz) Ultrashield spectrometer, and on a Bruker 500 MHz-Avance III (500 MHz) (Bruker, Billerica, MA, USA). Deuterated dimethylsulfoxide (DMSO-*d*_6_) and deuterated chloroform (CDCl_3_) were used as solvents in sample preparation, unless otherwise indicated. The chemical shifts were reported in ppm relative to tetramethylsilane (TMS), which was used as an internal reference standard.^1^H-NMR data are reported as follows: chemical shift (ppm), multiplicity, coupling constant (Hz), number of protons, the corresponding proton(s). Infrared (IR) spectra were recorded using a Shimadzu 8400F FT-IR spectrophotometer (Shimadzu, Kyoto, Japan). The samples were prepared as potassium bromide (KBr) (Sigma, St. Luis, MO, USA) disks. High-resolution mass spectra (HRMS) were measured in positive ion mode using electrospray ionization (ESI) technique by collision-induced dissociation on a Bruker APEX-4 (7 Tesla) instrument. The samples were dissolved in acetonitrile, diluted in spray solution (methanol/water 1:1 *v*/*v* + 0.1 M formic acid) and infused using a syringe pump with a flow rate of 2µL/min. External calibration was conducted using arginine cluster in a mass range *m*/*z* 175–871. Low-resolution mass spectra (LRMS) were measured by Applied Biosystems-MDS SCIEX API 3200 LC/MS/MS system (Applied Biosystems, Foster City, CA, USA), employing the positive mode using an electrospray ionizer (ESI) which was operated at 5.0–5.5 kV, with the capillary heater at 350 °C, sheath gas pressure 45 psi and an ion trap analyzer. Molecular weight was recorded as atomic mass unit (AMU) + 1, as the positive mode of ESI adds 1 AMU to the molecular ion peak. Some compounds were recorded as AMU + 2, as the positive mode of ESI adds 1 AMU to the molecular ion peak and so does the iontrap analyzer.

#### 3.1.1. Synthesis of Novel Compounds **3b** and **3c**

##### Synthesis of Compound **1**

Compound **1** was previously reported by our group [18]. It was synthesized following the same procedure, with slight modification.

##### Synthesis of New Compounds (Scheme 1)

*Ethyl1-cyclopropyl-6-fluoro-7-[(4-methyl phenyl)amino]-8-nitro-4-oxo-1,4-dihydroquinoline-3-carboxylate* (**2b**) (Scheme 1). Three equivalents of *p*-toluidine (0.9 g, 8.4 mmol) were added into a solution containing ethyl7-chloro-1-cyclopropyl-6-fluoro-8-nitro-4-oxo-1,4-dihydroquinoline-3-carboxylate (1.1 g, 2.8 mmol) and 10 mL of DMSO as a solvent and drops of pyridine, and was then refluxed at 65–70 °C under anhydrous conditions for 4–5 days. The reaction mixture was monitored until no starting material remained, then was left to crystallize at room temperature, and the product was filtered, soaked with petroleum ether, and left to dry in a dark place. Color of solid compound: orange; yield ≈ 50% (0.6 g); mp: 227–230 °C (decomposition); *R*_f_ value in system 2 = 0.54. ^1^H-NMR (300 MHz, DMSO-*d*_6_): 0.97 (m, 4H, H2-2′, H2-3′), 1.26 (t, *J* = 7.2 Hz, 3H, CH_3_CH_2_), 2.21(s, 3H, Ar-CH_3_), 3.58 (m,1H, H-1′), 4.22 (q, *J* = 7.2 Hz, 2H, CH_2_CH_3_), 6.85 (dd, *J* = 2 Hz, 8.0 Hz, 2H, H-2″, H-6″), 7.01 (dd, *J* = 2 Hz, 8.1 Hz, 2H, H-3″, H-5″), 8.00(d, 3*J*_H-F_ = 11.7 Hz, 1H, H-5), 8.54 (s, 1H, H-2), 8.60 (br s, 1H, NH, exchangeable).^13^C-NMR (75 MHz, DMSO-*d*_6_): 10.72 (C-2′,C-3′), 14.91 (CH_3_CH_2_), 21.0 (Ar-CH_3_), 39.15 (C-1′), 60.89 (CH_2_CH_3_), 112.11 (C-3), 115.48 (d, 2*J*_C-F_ = 21.5 Hz, C-5), 119.02 ( C-2″, C-6″), 124.15 (d, 3*J*_C-F_ = 6 Hz, C-4a), 129.85 (C-3″, C-5″), 131.72 (d, 2*J*_C-F_ = 16 Hz, C-7), 132.04 (C-8a), 133.43 (C-8), 135.89 (C-1″), 140.41 (C-4″), 151.99 (C-2), 152.83 (d, 1*J*_C-F_ = 258 Hz, C-6), 164.30 (CO_2_Et), 170.90 (d, 4*J*_C-F_ = 2 Hz, C-4). IR (KBr): ν 3430, 3346, 1731, 1625, 1523, 1460, 1319, 1180, 1029 cm^−1^. HRMS (ESI, +ve): calc. for C_22_H_21_FN_3_O_5_ [M + H]^+^ (426.13870). Found 426.14598. Anal. Calcd for C_22_H_20_FN_3_O_5_ (425.41): C, 62.11; H, 4.74; N, 9.88 Found C, 62.45; H, 5.13; N, 9.72.

*Ethyl 1-cyclopropyl-6-fluoro-7-[(4-methoxyphenyl)amino]-8-nitro-4-oxo-1,4-dihydroquinoline-3-carboxylate* (**2c**) (Scheme 1). Three molar equivalents of *p*-anisidine (3.1 g, 25.4 mmol) were added into a solution containing (1.3 g, 8.46 mmol) and 5–10 mL of DMSO as a solvent and drops of pyridine, and was then refluxed at 65–70 °C under anhydrous conditions for 2–3 days. The reaction mixture was monitored until no starting material remained, then was left to crystallize at room temperature. The product was filtered and left to dry in a dark place. Color of solid compound: red; yield ≈ 86% (3.2 g); mp: 245–247 °C (decomposition); *R*_f_ value in system 2 = 0.48. ^1^H-NMR (300 MHz, DMSO): 0.92, 0.95 (2 m, 4H, H2-2′, H2-3′), 1.25 (t, *J* = 7.1 Hz, OCH_2_CH_3_), 3.56 (m,1H, H-1′), 3.68 (s, 3H, OCH_3_), 4.2 (q, *J* = 7.74 Hz, 2H, OCH_2_CH_3_), 6.8 (d, *J* = 8.88 Hz, 2H, H-2″, H-6"), 6.96 (d, *J* = 8.13 Hz, 2H, H-3″, H-5″), 7.92 (d, 3*J*H-F = 12.3 Hz, 1H, H-5), 8.52 (br s, 1H, NH, exchangeable), 8.61 (s, 1H, H-2). ^13^C-NMR (75 MHz, DMSO): 10.46 (2C, C-2′, C-3′), 14.72 (OCH_2_CH_3_), 39.09 (C-1′), 55.74 (OCH_3_), 60.7 (OCH_2_CH_3_), 111.91 (C-3), 114.44 (2C, C-2″, C-6″), 115.24 (d, 2*J*_C-F_ = 21.15 Hz, C-5), 121.51 (2C, C-3″, C-5″), 122.85 (C-4a), 132.43 (d, 2*J*_C-F_ = 14.8 Hz, C-7), 133.56 (C-8a), 135.35 (C-8), 135.38 (C-1″), 151.77 (C-2), 151.9 (d, 1*J*_C-F_ = 250.65 Hz, C-6), 155.95 (C-4″), 164.11 (CO_2_Et), 170.66 (C-4). IR (KBr): ν 3425, 3363, 1728, 1610, 1500, 1435, 1327, 1080, 1033 cm^−1^. HRMS (ESI, +ve): calc. for C_22_H_20_FN_3_NaO_6_ [M + Na]^+^ (464.12338). Found 464.12283. LRMS (ES, +ve) *m*/*z* calc. for C_21_H_24_FN_3_O_5_ (441.13): Found 443.4 (88%, M+2), 442.0 (100%, M+1), 428.5 (2%), 412.6 (2%), 397.6 (7%), 396.6 (35%), 377.2 (1%), 335.2 (9%), 338.1 (1%), 322.1 (1%), 261.4 (1%), 102.1 (2%), 78.8 (1%), 74.3 (5%), 60.8 (1%), 59.2 (8%). Anal. Calcd for C_22_H_20_FN_3_O_5_ (441.41): C, 59.86; H, 4.57; N, 9.52 Found C, 60.04; H, 4.32; N, 9.85.

*1-Cyclopropyl-6-fluoro-7-[(4-methylphenyl)amino]-8-nitro-4-oxo-1,4-dihydroquinoline-3-carboxylic acid* (**3b**) (Scheme 1). A vigorously stirred suspension of ethyl 1-cyclopropyl-6-fluoro-7-[(4-methyl phenyl)amino]-8-nitro-4-oxo-1,4-dihydroquinoline-3-carboxylate (**2b**, 0.28 mmol, 0.12 g) was dissolved in a mixture of absolute ethanol (2 mL) and 12 N HCl (5 mL) under reflux at 80–90 °C for 24–48 h, and the reaction was monitored by TLC. At the end of the reaction, the reaction mixture was poured on crushed ice to precipitate a pure product that was collected by filtration and left to dry at room temperature. Color of solid compound: bright orange; yield ≈ 55.4% (0.062 g); mp: 243–245 °C. ^1^H-NMR (300 MHz, CDCl_3_): 0.87,1.14 (2 m, 4H, H2-2′, H2-3′), 2.29 (s, 3H, Ar-CH_3_), 3.69 (m, 1H, H-1′), 6.90 (d, *J* = 75 Hz, 2H, H-2″, H-6″), 7.09 (d, *J* = 7.8 Hz, 2H, H-3″, H-5″), 8.00 (br s, 1H, NH, exchangeable), 8.12 (d, 3*J*H-F = 12 Hz, 1H, H-5), 8.81 (s, 1H, H-2), 14.50 (br s, 1H, COOH). ^13^C-NMR (75 MHz, DMSO-*d*_6_): 10.54 (C-2′, C-3′), 20.9 (CH_3_), 40.4 (C-1′), 109.4 (C-3), 114.7 (d, 2*J*_C-F_ = 21.83 Hz, C-5), 119.2 (C-4″), 120.0 (C-2″, C-6″), 120.6 (d, 3*J*C-F= 7.8 Hz, C-4a), 129.7 (C-3″, C-5″), 133.0 (C-8a), 133.5 (d, 2*J*_C-F_ = 15.4 Hz, C-7), 134.3 (C-8), 139.4 (C-1″), 152.5 (C-2), 152.8 (d, 1*J*_C-F_ = 253 Hz, C-6), 165.4 (COOH), 175.8 (C-4). IR (KBr): ν 3433, 3363, 3063, 2924, 1728, 1620, 1519, 1458, 1319, 1242, 1103 cm^−1^. HRMS (ESI, +ve): calc. for C_20_H_16_ FN_3_O_5_ [M + H]^+^ (397.10740). Found 397.10012. Anal. Calcd for C_20_H_16_FN_3_O_5_ (397.36): C, 60.45; H, 4.06; N, 10.57 Found C, 60.86; H, 4.41; N, 10.79.

*1-Cyclopropyl-6-fluoro-7-(4-methoxy-phenylamino)-8-nitro-4-oxo-1,4-dihydro-quinoline-3-carboxylic acid* (**3c**) (Scheme 1). A vigorously stirred suspension of (**2c**, 0.6 g, 1.4 mmol) was dissolved in 12 mL mixture ofabsolute ethanol and 12 N HCl under reflux at 80–90 °C for 24–48 h, and the reaction was monitored by TLC. At the end of the reaction, the reaction mixture was poured on crushed ice to precipitate a pure product that was collected by filtration and left to dry at room temperature. Color of product: deep orange; yield ≈ 69% (0.4 g); mp: 210–215 °C; *R*_f_ value in system 1 = 0.5. ^1^H-NMR (300 MHz, DMSO-*d*_6_): 1.03, 1.21 (m, 4H, H2-2′, H2-3′), 3.62 (m, 1H, NCH-1′), 3.74 (s, 3H, OCH_3_ ), 7.05(d, *J* = 8.7 Hz, 2H, H-2″, H-6″), 7.36 (d, *J* = 8.8 Hz, 2H, H-3″, H-5″ ), 7.96 (d, 3*J*_H-F_ = 11.7 Hz, 1H, H-5), 8.56 (s, 1H, H-2), 8.85 (br s, 1H, NH, exchangeable), 14.66 (br s, 1H, COOH). ^13^C-NMR (75 MHz, DMSO-*d*_6_): 10.43 (2C, C-2′,C-3′), 39.85 (C-1′), 55.73(OCH_3_), 106.84 (d, 2*J*_C-F_ = 19 Hz, C-5), 109.35 (C-3), 114.13 (2C, C-2″, C-6″), 115.13 (2C, C-3″, C-5″), 122.65 (C-4a), 124.78 (C-8a), 132.45 (C-8), 134.46 (C-1″), 140.33 (C-7), 148.01 (C-2), 152.3 (d, 1*J*_C-F_ = 260 Hz, C-6), 156.65 (C-4″), 166.59 (COOH), 176.45 (C-4). IR (KBr): ν 3363, 3063, 2924, 2854, 1728, 1627, 1512, 1458, 1319, 1242, 1033 cm^−1^. HRMS (ESI +ve): calc. for C_20_H_17_FN_3_O_6_ [M + H]^+^ (414.11014). Found 414.10959. LRMS (ESI +ve): calc. for C_20_H_17_FN_3_O_6_ (413.1) Found 414.4 (5%, M + 1), 412.9 (76%), 384.2 (29%), 379.8 (1%), 367.4 (100%), 365.3 (13%), 361.3 (9%), 339.2 (58%), 331.7 (8%), 324.6 (15%), 321.2 (9%), 282.0 (3%), 240.9 (7%), 225.0 (4%), 217.4 (2%), 187.2 (1%), 145.0 (2%), 130.3 (13%), 123.4 (7%), 107.1 (1%), 101.0 (48%), 78.9 (93%), 74.5 (7%), 63.9 (13%), 59.0 (28%). Anal. Calcd for C_20_H_16_FN_3_O_6_ (413.36): C, 58.11; H, 3.80; N, 10.17 Found C, 57.86; H, 4.11; N, 10.55.

### 3.2. Microbiological Methods and Anti-Microbial Assays

#### 3.2.1. Bacterial Strains and Growth Conditions

Twelve *H. pylori* clinical isolates and one reference strain of *H. pylori* (NCTC 11916) were used in this study. The clinical strains were isolated from gastric biopsy samples obtained by a gastroenterologist of the Jordan University Hospital during a routine endoscopy. The gastric biopsy material was processed according to the standard methodology. Briefly, each biopsy for culture was homogenized using a tissue homogenizer (IKA, Staufen, Germany). Aliquots of 100 µL of the homogenate were primarily cultured on Columbia blood agar (Oxoid, Hampshire, UK) supplemented with 7% (*v*/*v*) horse blood and Dent selective supplement (Oxoid, Hampshire, UK). Subcultures of the bacteria were performed using the same plates but without the Dent supplement. All of the plates were incubated at 37°C under microaerophilic conditions using CampyGen atmosphere generating system (Oxoid, Hampshire, UK) in anaerobic jars for 5–7 days. Growth of *H. pylori* was confirmed according to colony morphology, Gram staining, microaerophilic growth (at 37 °C), biochemical tests (positive for oxidase, catalase, and urease), and subsequently by standard PCR of 16S ribosomal DNA test [21]. *H. pylori* cultures were stored at −70 °C in Trypticase soy broth (Oxoid, Hampshire, UK) containing 10% (*v*/*v)* fetal calf serum (PAA, Pasching, Austria) and 15% glycerol.

#### 3.2.2. Antimicrobial Susceptibility Testing and Minimal Inhibitory Concentration Determination

Bacterial suspensions were prepared to the 2 McFarland’s standard and subsequently uniformly spread on a solid growth medium in a Petri dish. Sterile paper disks (6 mm in diameter; Oxoid, Hampshire, UK) were impregnated with 25 μL of each compound, and were placed on the surface of each agar plate. Plates were incubated for 5–7 days under appropriate cultivation conditions. Antibacterial activity was determined by the production of an inhibition zone around the impregnated disc with the compounds. Disks impregnated with DMSO served as negative controls, and disks with standard antibiotics (ciprofloxacin and metronidazole, Oxoid, Hampshire, UK) served as positive controls. The minimal inhibitory concentrations (MICs) of each extract were determined by the two-fold agar dilution method as previously described [22]. In brief, each compound was serially diluted in DMSO and incorporated to molten Columbia blood agar plates supplemented with 7% (*v*/*v*) horse blood. Spot of *H. pylori* (1 × 10^5^ CFU) was applied on the surface of each plate, and the growth of visible colonies was determined after 7 days of incubation at 37 °C under microaerophilic conditions.

MIC was recorded as the lowest concentration that inhibited the visible growth of organisms; the plates with the standard antibiotics served as positive controls, and plates with DMSO served as negative controls. Triplicates of each tested compound were performed, and the average of the results was taken.

#### 3.2.3. Determination of in Vitro Interaction

Antimicrobial interactions between each compound and metronidazole against three strains (two clinical isolates and one reference strain) of *H. pylori* were evaluated by the standard checkerboard titration method [23]. Each compound and metronidazole was dissolved in DMSO and distilled water, respectively. The bacterial inoculum, media, and culture conditions were the same as those described for the MIC determination mentioned above. Experiments were performed in triplicate.

The fractional inhibitory concentrations (FICs) were calculated as follows:

- FIC = (MIC of drug A in combination/MIC of drug A alone) + (MIC of drug B in combination/MIC of drug B alone).
- The FIC indices were interpreted as follows: ≤0.5, synergy; 0.5–1, additive; 1–4.0, indifference; >4.0, antagonism.

### 3.3. Urease Inhibition Assay

Urease inhibition was performed as described elsewhere [24]. Briefly, 10 μL of 1 × 10^8^ CFU/mL *H. pylori* suspension was incubated with equal amount of serially diluted compound in 96-well microplates for 30 min at 37 °C. Subsequently, 200 μL of detection reagent composed of 50 mM phosphate buffer, pH 6.8 containing 500 mM urea and 0.02% phenol red was added to each well. The color development was measured at 555 nm in 5 min intervals. Controls included bacteria with the reagent, and reagent with each compound. The percentage of inhibition was calculated by the equation % inhibition = [(activity without compound − activity with compound)/(activity without compound)] × 100. The activity was compared to a reference urease inhibitor—acetohdyroxamicacid (Sigma-Aldrich, St. Louis, MI, USA).

## 4. Conclusions

In conclusion, this work successfully introduced new 8-nitro-fluoroquinolone derivatives utilizing a new procedure developed within the course of this work. The fluoroquinolone derivatives were fully identified and characterized using NMR, IR, electrochemical analysis (EA), and MS. The antimicrobial properties of all pure compounds were evaluated against *H. pylori*. Although the selected structures did not follow a specific pattern (structure), it could be suggested that different hydrophilic/lipophilic properties and different intra-cellular targets are considered for further investigation of the mechanism of antibacterial action

The results clearly indicate that all new compounds have interesting antimicrobial activity against metronidazole-resistant *H. pylori*. In addition, these compounds could be considered good candidates for studying the combination effect with metronidazole in vivo for future potential as alternatives in the triple therapy regimen used in clinical practice.

## Figures and Tables

**Table 1:** Zones of inhibition (mm) of synthesized compounds (1 mg/mL), ciprofloxacin, and metronidazole against clinical strains of *H. pylori* (**1**–**12**) and control strain.

Tested Compound	Zones of Inhibition (mm)												
	Clinical Isolates												Control Strain
	1	2	3	4	5	6	7	8	9	10	11	12	
**3a**	10	20	15	10	20	11	11	17	9	15	13	15	18
**3b**	12	12	0	15	11	16	13	15	11	12	14	13	12
**3c**	20	24	20	16	24	20	18	25	15	20	23	25	25
**3d**	10	10	8	12	13	11	14	15	9	14	13	12	12
**3e**	10	12	0	15	15	14	12	16	9	15	15	14	13
CIP	20	40	0	40	50	45	50	0	0	45	50	45	45
MTZ	10	0	0	0	0	10	0	0	0	0	0	0	0
DMSO	0	0	0	0	0	0	0	0	0	0	0	0	0

CIP: ciprofloxacin; MTZ: metronidazole.

**Table 2:** Minimum inhibitory concentration (MIC, µg/mL) of tested compounds and metronidazole against clinical strains of *H. pylori* (**1**–**12**) and control strain by two-fold agar dilution method.

Compounds	Minimum Inhibitory Concentration (µg/mL)												
	Strain Number												Control Strain
	1	2	3	4	5	6	7	8	9	10	11	12	
**3a**	32	16	16	16	8	32	16	8	16	32	16	16	16
**3b**	32	32	16	32	32	64	32	32	32	32	32	32	32
**3c**	4	4	8	4	2	4	4	8	4	4	8	4	8
**3d**	32	16	16	16	32	32	16	16	32	32	16	32	16
**3e**	16	32	32	16	32	32	16	32	32	32	16	32	32
CIP	0.3	0.6	0.3	0.6	0.3	0.04	0.6	0.04	0.08	0.04	0.6	0.6	0.6
MTZ	64	64	64	32	128	64	128	32	64	64	128	64	128

**Table 3:** Fractional inhibitory concentration (FIC) values of tested compounds in combination with metronidazole against clinical strains of *H. pylori* (**11**, **12**) and control strain by standard checkerboard titration method with two-fold serial agar dilutions.

Compounds	FIC Values (Index) (MIC Combination)			FIC Mean
	Strain Number			
	11	12	Control Strain	
3a-MTZ	0.328 (*)	0.328 (*)	0.328 (*)	0.328 (*)
	(2.5)	(2.5)	(2.5)	
3b-MTZ	1.312 (=)	1.312 (=)	1.312 (=)	1.312 (=)
	(10)	(10)	(10)	
3c-MTZ	0.656 (+)	0.656 (+)	0.656 (+)	0.656 (+)
	(5)	(5)	(5)	
3d-MTZ	0.328 (*)	0.328 (*)	0.328 (*)	0.328 (*)
	(2.5)	(2.5)	(2.5)	
3e-MTZ	1.312 (=)	0.656 (+)	0.656 (+)	0.874 (+)
	(10)	(5)	(5)	

(*): Synergism; (+): Additive; (=): Indifferent.

**Table 4:** Urease inhibition activity of the synthetic compounds. All compounds tested in the concentration range of 0.5–500 µg/mL. The effect was considered significant if the inhibition was more than 60%. Acetohydroximic acid (AHA) was used as positive control. NS = not significant.

Compound	IC_50_ (µM)
**3a**	NS
**3b**	629
**3c**	151
**3d**	NS
**3e**	NS
**AHA**	27
